# Complement-mediated and direct activation of human neutrophils induced by *Premolis semirufa* caterpillar toxins

**DOI:** 10.3389/fimmu.2025.1706235

**Published:** 2026-01-02

**Authors:** Joel J. M. Gabrili, Ângela A. A. Megale, Giselle Pidde, Trent M. Woodruff, Denise V. Tambourgi

**Affiliations:** 1 Immunochemistry Laboratory, Instituto Butantan, São Paulo, Brazil; 2 School of Biomedical Sciences, Faculty of Medicine, The University of Queensland, Brisbane, QLD, Australia

**Keywords:** pararamosis, joint disease, innate immunity, complement, neutrophils, inflammation, complement-targeted therapy

## Abstract

**Introduction:**

Pararamosis is an occupational inflammatory disease caused by contact with hairs of the caterpillar *Premolis semirufa*, endemic to the Brazilian Amazon and primarily affecting rubber tree workers. Exposure to pararama hairs induces acute pruritic dermatitis and, in some cases, chronic joint inflammation resembling rheumatoid arthritis and osteoarthritis. Previous studies demonstrated that pararama hair extract activates the complement system and induces a robust inflammatory response in mouse models, characterized by cytokine production and immune cell recruitment. Given the critical role of neutrophils in inflammation and their activation by complement components, this study investigated the effects of pararama hair extract on human peripheral blood neutrophils.

**Methods:**

Neutrophils isolated from healthy donors were directly stimulated with the extract or incubated with plasma derived from whole blood treated with the extract, in the presence or absence of compstatin, a C3 inhibitor that blocks complement activation, and PMX205, a selective C5aR1 antagonist applied directly to neutrophils. We assessed neutrophil activation by measuring cytokine and chemokine secretion, myeloperoxidase and elastase release, and neutrophil extracellular trap (NET) formation.

**Results:**

Our results reveal that pararama hair extract directly activates neutrophils, enhancing pro-inflammatory mediator release and degranulation. Moreover, plasma from extract-treated human whole blood samples further potentiated neutrophil activation, which was significantly reduced by compstatin, through inhibition of C3, and by selective blockade of the C5a receptor (C5aR1).

**Discussion:**

These findings highlight the integrated role of complement in neutrophil activation, as compstatin broadly inhibited complement-dependent effects, while PMX205 demonstrated the specific contribution of the C5a–C5aR1 axis. Targeting complement thus emerges as a promising strategy to mitigate inflammation and tissue damage in affected individuals.

## Graphical Abstract

**Figure:**
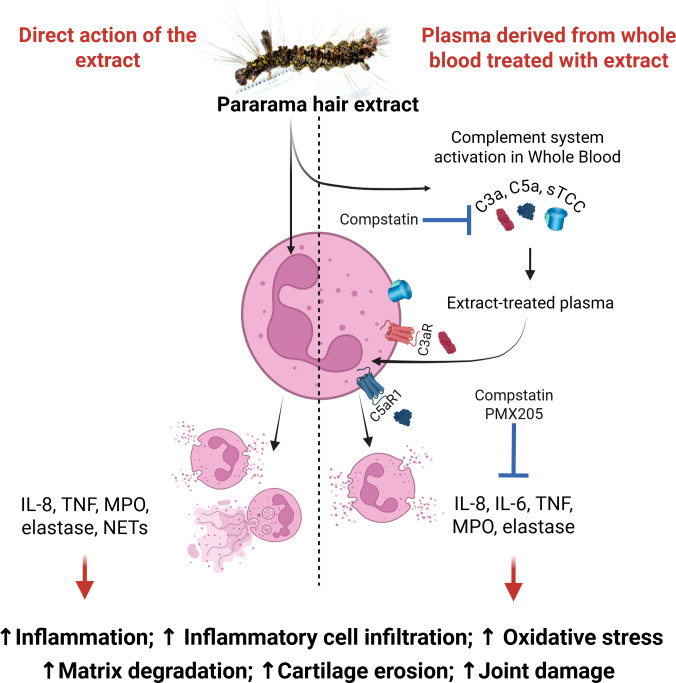
Illustrating two mechanisms of neutrophil activation by the hair extract of the *Premolis semirufa* caterpillar (Pararama): a direct effect of the extract on neutrophils, and an indirect effect mediated by plasma derived from extract-treated whole blood, through complement activation. On the left, the extract directly induces the production of inflammatory mediators (IL-8, TNF-α, elastase, MPO) and the formation of NETs. On the right, the extract, when incubated with whole blood, activates the complement system, leading to the generation of C3a, C5a, and sTCC. Plasma derived from this activation stimulates neutrophils to produce IL-8, IL-6, TNF-α, elastase, and MPO. The action of complement inhibitors is represented by blue inhibitory lines: compstatin (C3 inhibitor), which prevents complement activation and thereby the generation of C3a, C5a, and sTCC; and PMX205 (C5aR1 antagonist), which blocks C5a–C5aR1 signaling on neutrophils. Both interventions reduce neutrophil outputs (IL-8, IL-6, TNF-α, elastase, MPO). In both contexts, activated neutrophils contribute to inflammation, cellular infiltration, oxidative stress, matrix degradation, cartilage erosion, and joint damage. Moreover, inhibition by compstatin and PMX205 attenuates these inflammatory and degenerative effects. Red arrows indicate inflammatory outcomes, whereas blue inhibitory lines represent inhibitory/anti-inflammatory actions. Created with BioRender.com.

## 1 Introduction

Pararamosis is an occupational disease characterized by an inflammatory response induced by contact with the hairs of the caterpillar or cocoon of the moth *Premolis semirufa* (1). The term “pararamosis” derives from the common name of the larval form of this species, known locally as “pararama”. This caterpillar is native to the Brazilian Amazon region and inhabits rubber trees (Hevea spp.), where it feeds on the foliage (2).

The initial reports of pararamosis emerged during the 1940s among rubber plantation workers in a region of the Brazilian Amazon in the state of Pará, with up to 40% of laborers in a local village reportedly affected (3). Subsequent investigations enabled the identification of the etiological agent (4, 5). Although lesions may occur on various body regions, the hands are most frequently affected due to the occupational nature of exposure, typically presenting as pruritic dermatitis following contact with larval hairs (1, 6).

Exposure to the hairs triggers immediate reactions characterized by intense pruritus, followed by pain, redness, and localized heat, hallmarks of an acute inflammatory response lasting between 3 to 7 days (1). However, some individuals may develop chronic inflammation, resulting in thickening of the articular synovial membrane and potential progression to joint deformities and immobilization. This clinical presentation of ankylosis shares certain features with both rheumatoid arthritis (RA) (2, 4) and osteoarthritis (OA) (7, 8). Despite the absence of a specific treatment, corticosteroids have been used to mitigate the progression or alleviate the symptoms associated with the chronic form of the disease (9, 10).

Our group, addressing the public health impact and lack of treatments for pararamosis, found that Pararama caterpillar hair extract contains enzymes, including hyaluronidase, serine- and metalloproteases, that act synergistically to contribute to disease symptoms (10, 11). In mouse models, repeated exposure caused strong local inflammation with neutrophil infiltration, macrophage recruitment, and elevated cytokines including IL-1, IL-2, IL-4, IL-6, IL-10, IL-12, IL-17, IL-23, IFN-γ, and TNF-α (11, 12). The extract activates the human complement system, producing anaphylatoxins and the soluble terminal complement complex (sTCC) (10). In whole blood, it stimulates monocytes and granulocytes, increasing immune receptors such as CD11b, CD14, TLR2, TLR4, and C5aR1, and induces cytokines and chemokines including IL-17, TNF-α, CCL2, CCL5, CXCL8, CXCL9, and CXCL10, with these responses diminished by complement inhibition. Additionally, in endothelial cells, the extract promotes secretion of IL-8 and MCP-1, also reduced by complement inhibitors (13). These findings underscore the role of the extract in complement-mediated inflammation, contributing to the pathophysiology of pararamosis and highlighting potential therapeutic targets. Further investigations demonstrated its deleterious effects on joint-resident cells, triggering inflammatory and catabolic responses consistent with chronic joint diseases. Exposure of human chondrocytes to the extract enhanced production of IL-6, IL-8, MCP-1, prostaglandin E2, various MMPs, and complement components, while downregulating cartilage markers like collagen II and aggrecan (8). Proteomic analysis of the Pararama bristle extract, integrated with transcriptomic data from bristle-producing tissues, also identified proteins with significant homology to human molecules involved in inflammatory responses, extracellular matrix organization and innate immune pathways implicated in the pathogenesis of osteoarthritis and rheumatoid arthritis (7). Additional studies in human chondrocytes confirmed activation of NF-κB, increase in inflammatory mediators, and downregulation of cartilage-specific genes, while co-cultures of chondrocytes and synoviocytes exposed to the extract showed elevated TNF-α, IL-6, IL-8, COX-2, and MMPs, supporting the arthritogenic potential of the extract relevant to pararamosis pathology (14, 15).

The complement system is a key component of innate immunity, consisting of over 50 soluble and membrane-bound proteins that function cooperatively to eliminate pathogens, regulate inflammation, and maintain tissue homeostasis. It can be activated through three distinct pathways (classical, lectin, and alternative), all converging at the cleavage of C3, leading to the generation of opsonins (C3b), inflammatory peptides (C3a, C5a), and the terminal complement complex (TCC/C5b-9) (16, 17). Activation is tightly regulated by a range of soluble and membrane-associated inhibitors, ensuring protection of host cells (18–20).

Among its effector functions, the anaphylatoxins C3a and C5a play pivotal roles in orchestrating the inflammatory response. C5a, in particular, is a potent chemoattractant and activator of neutrophils, enhancing adhesion, degranulation, reactive oxygen species (ROS) production, and cytokine release via interaction with its receptor C5aR1 (CD88) (21, 22). C3a also promotes immune cell recruitment and cytokine production through C3aR signaling, although its role is more modulatory (17, 23, 24). Dysregulated complement activation has been implicated in a wide spectrum of inflammatory and autoimmune disorders, including systemic lupus erythematosus, rheumatoid arthritis, and vasculitides (25–27). As such, therapeutic targeting of complement components and receptors, such as with C1 inhibitors, C3 blockers (e.g., compstatin), or C5aR1 antagonists (e.g., PMX205), has emerged as a promising strategy for controlling complement-mediated inflammation (28, 29).

Neutrophils are key effector cells of the innate immune system, critically involved in the initiation and maintenance of the inflammatory response. While they exhibit robust antimicrobial activity, their dysregulated activation can contribute to tissue injury (30–32). Neutrophil cytoplasm is enriched with granules containing a range of bioactive mediators, including myeloperoxidase (MPO) and neutrophil elastase (NE), which are central to their effector functions. These enzymes possess microbicidal properties, facilitate extracellular matrix degradation, and modulate immune cell activation, thereby playing a pivotal role in host defense and inflammatory pathogenesis (33–41).

Neutrophils can be activated by various ligands, including those that engage pattern recognition receptors (PRRs) and G-protein-coupled receptors (GPCRs). Among them, C5aR1 (CD88), a GPCR expressed on neutrophils, is activated by C5a and mediates key inflammatory responses such as chemotaxis, degranulation, reactive oxygen species production, and upregulation of CR3 and CR1. The C5a–C5aR1 axis plays a pivotal role in neutrophil recruitment and activation in inflammatory diseases like rheumatoid arthritis and psoriatic arthritis. It also contributes to ANCA-associated neutrophil activation and the formation of neutrophil extracellular traps (NETs), including in COVID-19-related immunopathology (42–48).

Given the pivotal role of neutrophils in the initiation and progression of inflammatory joint diseases, and their activation by various mediators, including complement activation products, we sought to investigate their responsiveness to pararama hair extract. We were further motivated by the notable accumulation of neutrophils at sites of extract inoculation in animal models, and by previous demonstrations of complement activation by the extract in human serum and whole blood. To this end, we assessed neutrophil activation following direct exposure to the extract, as well as after incubation with plasma derived from human whole blood, in the presence or absence of pharmacological inhibitors targeting specific components of the complement cascade.

## 2 Materials and methods

### 2.1 Pararama hair extract

*P. semirufa* caterpillars were collected in the city of São Francisco, State of Pará (PA), Brazil (authorization no. 45166-6). Caterpillar hairs were cut and transferred to tubes containing PBS buffer (8.1 mM Na2HPO4; 1.5 mM KH2PO4; 137 mM NaCl; 2.7 mM KCl; pH 7.4) and frozen at -80°C until its use. To prepare the extract, samples were macerated using a glass rod and then the insoluble material was removed by centrifugation at 1125 x g for 20 minutes at 4°C. The supernatant was collected, filtered in 0.22 µm sterilization membrane (Wathman - GE Healthcare, Illinois, USA), aliquoted and stored at -80°C (Genetic Heritage Access Registry n° AEA2993).

The protein concentration of the extract was determined by the bicinchoninic acid method (BCA-Protein Assay Kit, Thermo Scientific, NY, USA) and the reading was performed on a spectrophotometer (Multiskan EX, Labsystems, Helsinki, Finland) at 540 nm, according to the manufacturer’s recommendations. Endotoxin contamination in the pararama hair extract was assessed using the PYROGENT™ Plus Gel Clot LAL Assay (Lonza, MD, USA) by the Microbial Control Unit of the Butantan Institute (São Paulo, SP, Brazil). The analysis revealed endotoxin levels below the detection limit (0.125 EU/mL), confirming that the findings of this study were attributable to the effects of the pararama hair extract itself.

### 2.2 Isolation of neutrophils from peripheral blood

Neutrophils were isolated from the whole blood (5 mL) obtained by venipuncture from healthy donors (Human Research Ethics Committee from the University of São Paulo, São Paulo, Brazil, certificate number 1452/18) and collected in Falcon tubes containing 25 IU/mL of heparin (Cristália, São Paulo, Brazil). Erythrocytes were sedimented in 2.5 mL of BSS buffer (137 mM NaCl; 2.68 mM KCl; 78.3 mM Na_2_HPO_4_; 1.47 mM KH_2_PO_4_) supplemented with 6% Dextran 70 (Sigma-Aldrich, Missouri, USA) for 40 minutes at room temperature. Following sedimentation, the supernatant was collected, adjusted to 5 mL with BSS, and centrifuged at 710 x g for 2 minutes. The supernatant was discarded, and the pellet was resuspended in 1.5 mL of distilled water, vortexed briefly for 10 seconds to facilitate complete suspension and lysis of residual erythrocytes. To re-establish osmotic equilibrium, 5 mL of BSS was added before a second centrifugation at 710 x g for 2 minutes. The supernatant was again discarded, and the pellet was resuspended in 1 mL of Krebs-Hepes buffer (120 mM NaCl; 25 mM HEPES; 4.8 mM KCl; 1.2 mM KH_2_PO_4_; 1.2 mM MgSO_4_). The cell suspension was layered onto 5 mL of Ficoll-Paque PLUS (GE Healthcare, Illinois, USA) and centrifuged at 180 x g for 30 minutes. After centrifugation, supernatants were discarded, and the pellets were resuspended in 1 mL of BSS. Cell counts were conducted using a Neubauer chamber, and cell viability was evaluated using 0.4% Trypan blue. Neutrophil purity was analyzed by flow cytometry based on cell size (FSC – Forward Scatter) and complexity (SSC – Side Scatter).

### 2.3 Treatment of neutrophils with pararama hair extract

Neutrophils were plated at a density of 1x10^5^ cells per well and stimulated with DMEM medium without FBS or with different concentrations of pararama hair extract (15 µg/mL, 30 µg/mL, and 60 µg/mL) for 1, 2, and 4 hours in a CO_2_ incubator at 37°C. Following stimulation, supernatants were collected for cytokines (IL-2, IL-4, IL-6, IL-10, IL-17A, IFN-γ, and TNF-α) and chemokines (CXCL8/IL-8, CCL5/RANTES, CXCL9/MIG, CCL2/MCP-1, and CXCL10/IP-10) analysis by Cytometric Bead Array (CBA) kits (BD Biosciences, California, USA). Myeloperoxidase and elastase levels were quantified using the Human Myeloperoxidase (MPO) ELISA Kit and Human PMN Elastase ELISA Kit (ABCAM, Cambridge, UK), following manufacturer protocols. Trypan blue exclusion was used to assess cell viability after each treatment.

### 2.4 Treatment of neutrophils with plasma derived from whole blood

In parallel with neutrophil isolation, a whole blood assay was performed using Lepirudin-anticoagulated blood from the same donors. Blood samples were incubated for 30 minutes at 37°C with either PBS, pararama hair extract (95 µg/mL), or a combination of the extract (95 µg/mL) and compstatin (ICVVQDWGHHRCT, 50 µM) or a control peptide (IAVVQDWGHHRAT, 50 µM) (Tocris Bioscience, Bristol, United Kingdom). Plasma, devoid of EDTA, was obtained by centrifuging the blood at 404 x g for 10 minutes at 4°C post-incubation. These plasma samples were maintained at 4°C and used within 30 minutes. Subsequently, neutrophils (1×10^5^ cells per well) were stimulated with the following: plasma from blood treated with PBS (PBS-treated plasma), plasma from blood treated with the extract (extract-treated plasma), plasma from blood treated with the extract plus compstatin (extract/compstatin-treated plasma), or plasma from blood treated with the extract plus control peptide (extract/control peptide-treated plasma). All plasma treatments were diluted 1:10 in culture medium.

To elucidate the role of the anaphylatoxin C5a, neutrophils were pretreated for 10 minutes with either PMX205 (10 µM) or its dilution vehicle (5% glucose). These cells were then incubated with plasma derived from whole blood treated with PBS or the extract. Following 2- and 4-hour incubations at 37°C in a CO_2_ incubator, cell supernatants were harvested. The levels of cytokines, chemokines, myeloperoxidase, and elastase in the supernatants were then quantified. Trypan blue exclusion was used to assess cell viability after each treatment.

### 2.5 Quantification of cytokines and chemokines

Measurement of cytokines (IL-2, IL-4, IL-6, IL-10, IL-17A, IFN-γ and TNF-α) and chemokines (CXCL8/IL-8, CCL5/RANTES, CXCL9/MIG, CCL2/MCP-1 and CXCL10/IP-10) was performed using the BD™ Cytometric Bead Array (CBA) Human Inflammatory Cytokine - I Kit and BD™ Cytometric Bead Array (CBA) Human Chemokine Kit (BD Biosciences, California, USA), respectively, on supernatants of cultures treated as described above, following the manufacturer’s instructions.

### 2.6 Quantification of myeloperoxidase and elastase

Myeloperoxidase (MPO) and elastase were quantified by ELISA with the Human Myeloperoxidase ELISA and the Human PMN Elastase ELISA Kits (Abcam, Cambridge, UK), following the manufacturer’s instructions.

### 2.7 Quantification of NETs

To evaluate the generation of neutrophil extracellular traps (NETs) in the culture supernatant, isolated peripheral blood neutrophils (1×10^5^ cells/well) were treated with PBS, 60 µg/mL of pararama hair extract, or PMA (10 uM) for 2 hours at 37°C in an incubator with 5% CO_2_. After stimulation, the MPO-DNA PicoGreen assay was utilized, as outlined by (49–53). A 96-well clear-bottom black plate (Corning) was pre-coated with an anti-MPO antibody (1:1000, Thermo Fisher Scientific) and incubated overnight at 4°C. After a washing step, the wells were blocked with a 2% PBS-BSA solution for two hours at room temperature and subsequently incubated with neutrophil supernatants overnight at 4°C. DNA quantification was performed using the Quanti-iT PicoGreen Kit (Thermo Fisher Scientific), and fluorescence intensity was measured using the Cytation 5 system (BioTek Instruments, Inc., USA) with excitation at 488 nm and emission at 525 nm, following the manufacturer’s instructions. This methodology enabled the detection of MPO-DNA complex formation.

### 2.8 Statistical analysis

Data were expressed as mean ± standard error of the mean (SEM) and analyzed using GraphPad Prism software, version 10.6.1 (GraphPad Software, San Diego, CA, USA). Comparisons among groups were performed using one-way ANOVA. Differences were considered statistically significant at *p* < 0.05.

## 3 Results

### 3.1 Direct effect of pararama hair extract on peripheral blood neutrophils

Neutrophils were isolated from peripheral blood according to the protocol described in the Materials and Methods. The purity of the isolated neutrophil population was verified by flow cytometry, demonstrating an approximate purity of 97% (**Supplementary Figure 1**). To evaluate the effect of pararama hair extract on neutrophil activation, cells were incubated with concentrations of 15 µg/mL, 30 µg/mL, and 60 µg/mL of the extract for 1, 2, and 4 hours. Subsequently, the levels of cytokines, chemokines, myeloperoxidase, and elastase released by neutrophils were measured in the culture supernatants.

**Figure 1:**
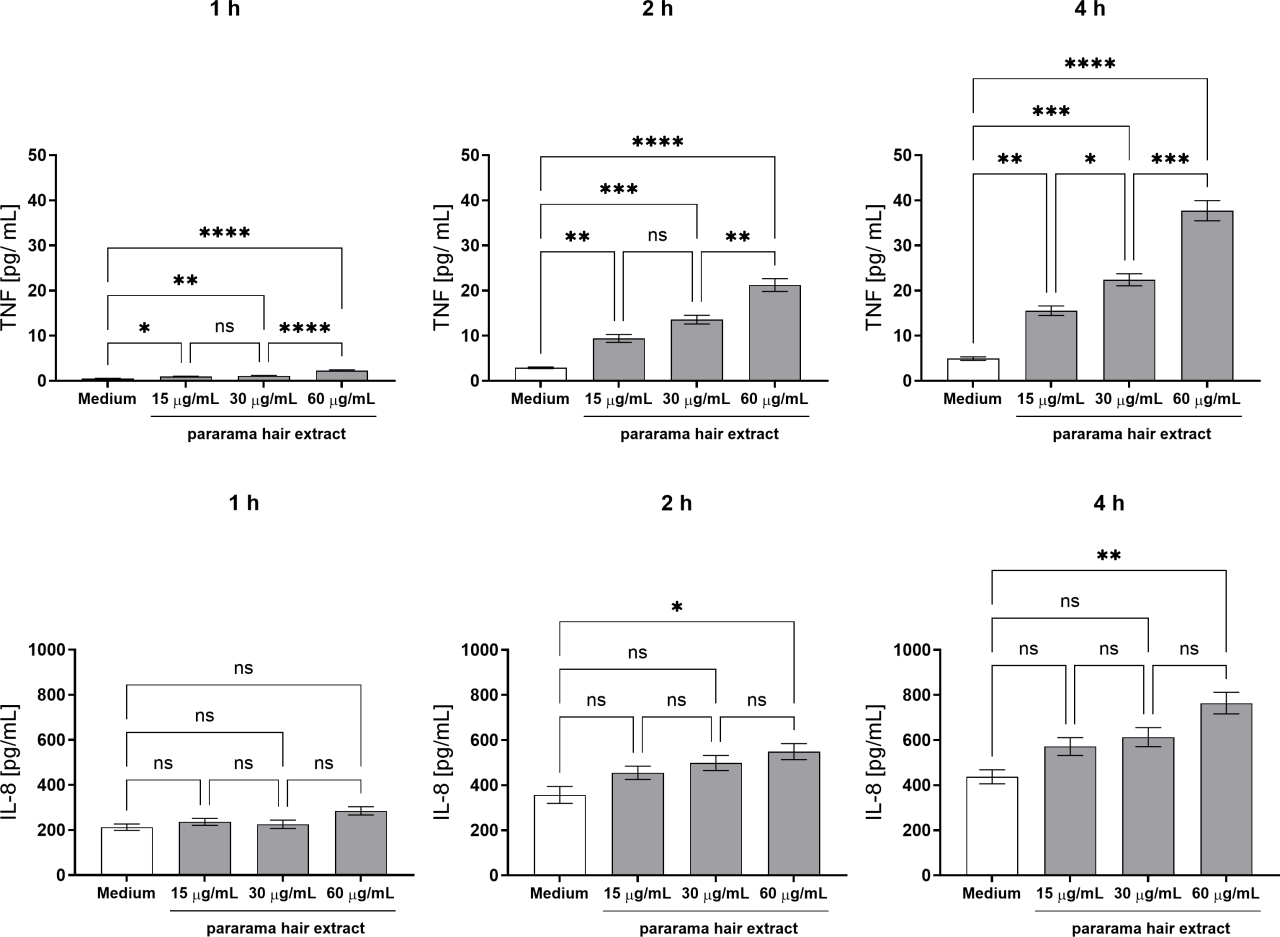
TNF-α and IL-8 Production by Human Neutrophils Stimulated with *Premolis semirufa* Hair Extract. Neutrophils isolated from peripheral blood (1×10^5^ cells/well) were treated with 15 µg/mL, 30 µg/mL, or 60 µg/mL of pararama hair extract or culture medium for 1, 2, and 4 h at 37°C in an incubator with 5% CO_2_. After stimulation, TNF-α and CXCL8/IL-8 production was determined in the culture supernatants using the Cytometric Bead Array. Results are presented as mean ± SEM of triplicates from three independent experiments. *p < 0.05, **p < 0.01, ***p < 0.001 and ****p < 0.0001.

As shown in **Figure 1**, TNF-α was detectable at all concentrations of pararama hair extract as early as 1 hour and remained significantly elevated at 2 and 4 hours. IL-8 levels showed no significant changes at 1 hour across any concentration. At 2 and 4 hours, IL-8 production increased only at the highest extract concentration (60 µg/mL). Conversely, the extract did not elicit measurable production of other cytokines (IL-2, IL-4, IL-6, IL-10, IL-17, IFN-γ) or chemokines (CCL5/RANTES, CXCL9/MIG, CCL2/MCP-1, CXCL10/IP-10) at any evaluated time point (data not shown).

**Figure 2:**
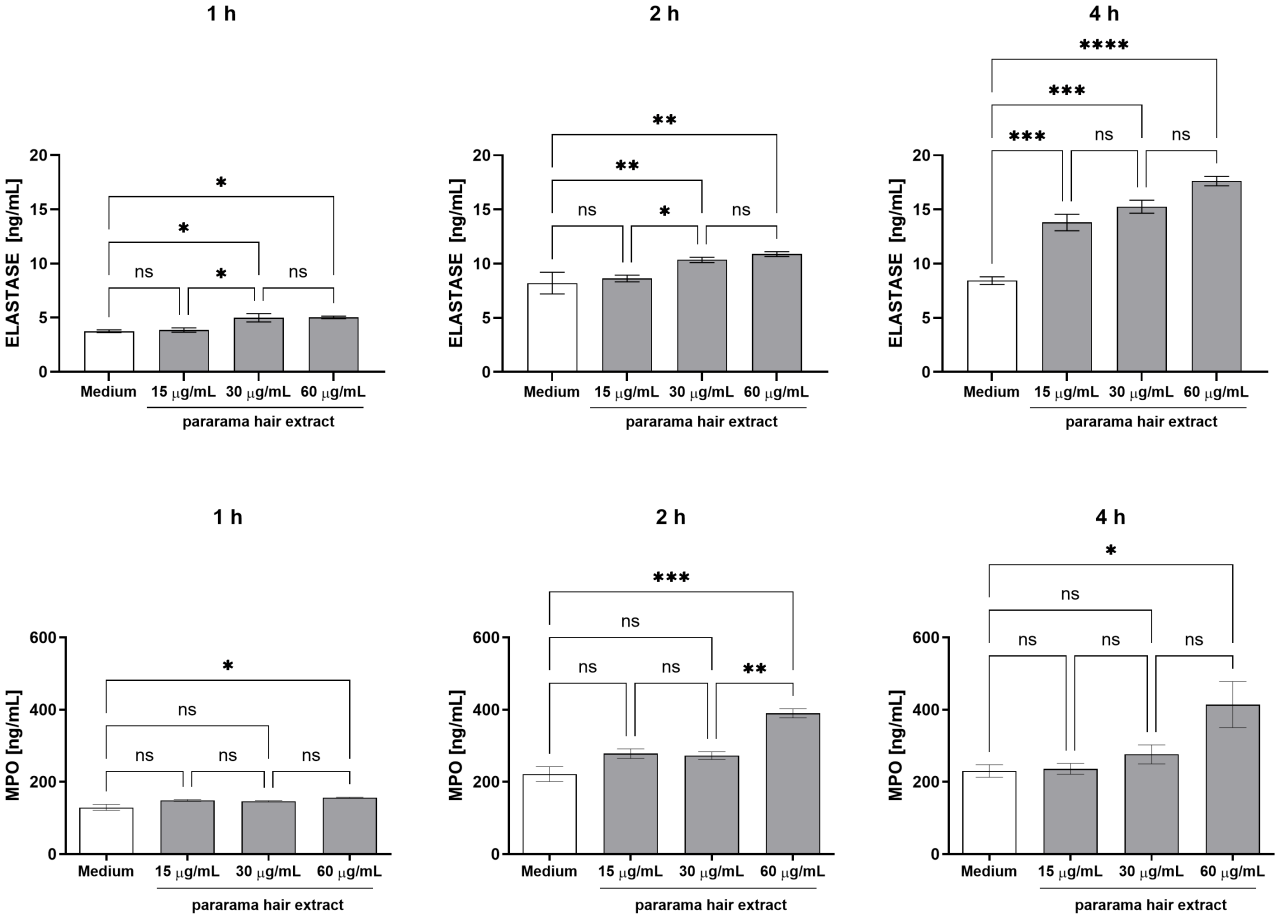
Myeloperoxidase and Elastase Production by Human Neutrophils Stimulated with *P. semirufa* Hair Extract. Neutrophils isolated from peripheral blood (1×10^5^ cells/well) were treated with 15 µg/mL, 30 µg/mL, or 60 µg/mL of pararama hair extract or culture medium for 1, 2, and 4 h at 37°C in an incubator with 5% CO_2_. After stimulation, myeloperoxidase (MPO) and elastase production was determined in the culture supernatants by ELISA. Results are presented as mean ± SEM of triplicates from three independent experiments. *p < 0.05, **p < 0.01, ***p < 0.001, ****p < 0.0001.

Neutrophil exposure to pararama hair extract also triggered the release of elastase, detectable after 1 hour at concentrations of 30 µg/mL and above. At 2 hours, elastase release was sustained at both 30 and 60 μg/mL, while by 4 hours, it was induced across all concentrations tested (**Figure 2**). Myeloperoxidase (MPO) production was observed starting at 1 hour with the highest extract concentration (60 μg/mL), persisting through the 2- and 4-hour time points.

**Figure 3:**
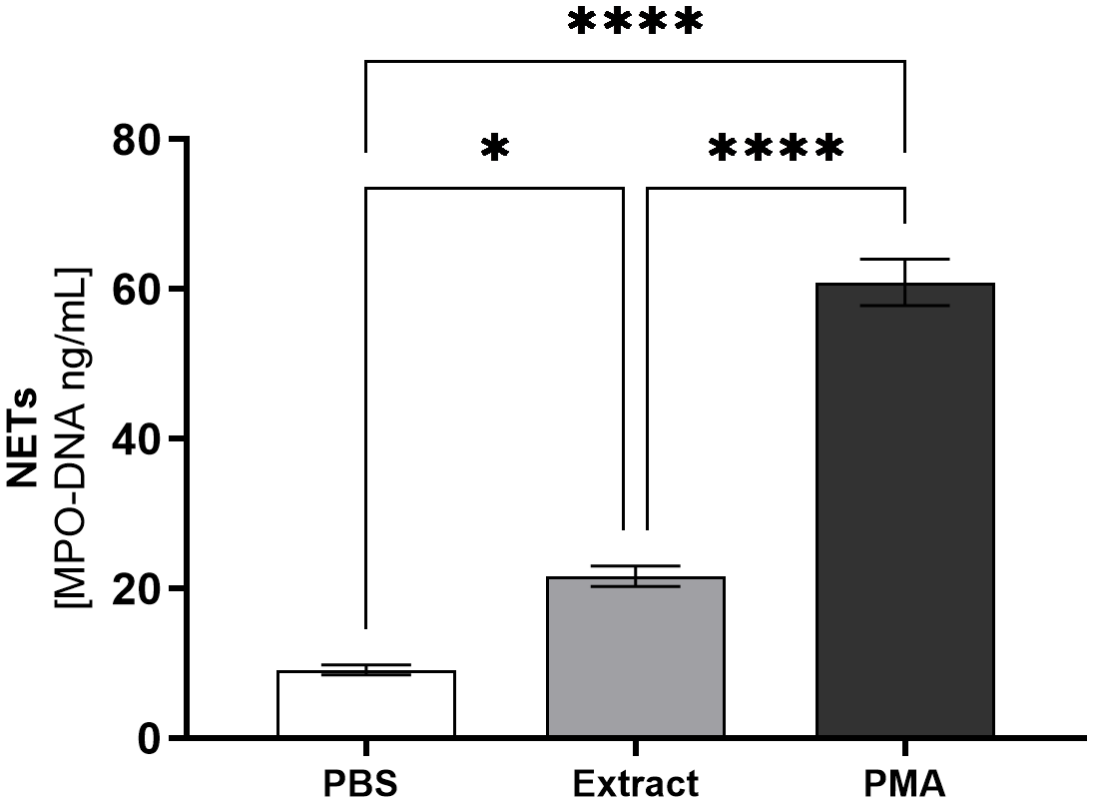
NETs (MPO-DNA Complex) by Human Neutrophils Stimulated with *P. semirufa* Hair Extract. Neutrophils isolated from peripheral blood (1×10^5^ cells/well) were treated with PBS, 60 µg/mL of pararama hair extract, or PMA (10 µM) for 2 h at 37°C in an incubator with 5% CO_2_. After stimulation, MPO-DNA complex formation was determined in the culture supernatants using the PicoGreen assay. Results are expressed as mean ± SEM of triplicates from three independent experiments. *p < 0.05, ****p < 0.0001.

Neutrophil extracellular trap (NET) formation was assessed by measuring MPO-DNA complexes in culture supernatants. As illustrated in **Figure 3**, stimulation with pararama hair extract significantly increased NET release compared to the negative control (PBS), although the levels remained lower than those induced by the positive control (PMA).

### 3.2 Effect of plasma derived from whole blood treated with pararama hair extract on neutrophils

Neutrophils are highly responsive cells that can be activated by various stimuli, including products derived from the complement system (21, 48, 54). To evaluate whether mediators produced by the extract in whole blood could stimulate neutrophils *in vitro*, we incubated neutrophils with plasma obtained from whole blood samples treated with the extract and measured cytokine, chemokine, MPO, and elastase levels in the culture supernatants. To investigate the role of complement system activation products generated by the extract in the neutrophil response, the C3 inhibitor compstatin and its control peptide were used.

**Figure 4:**
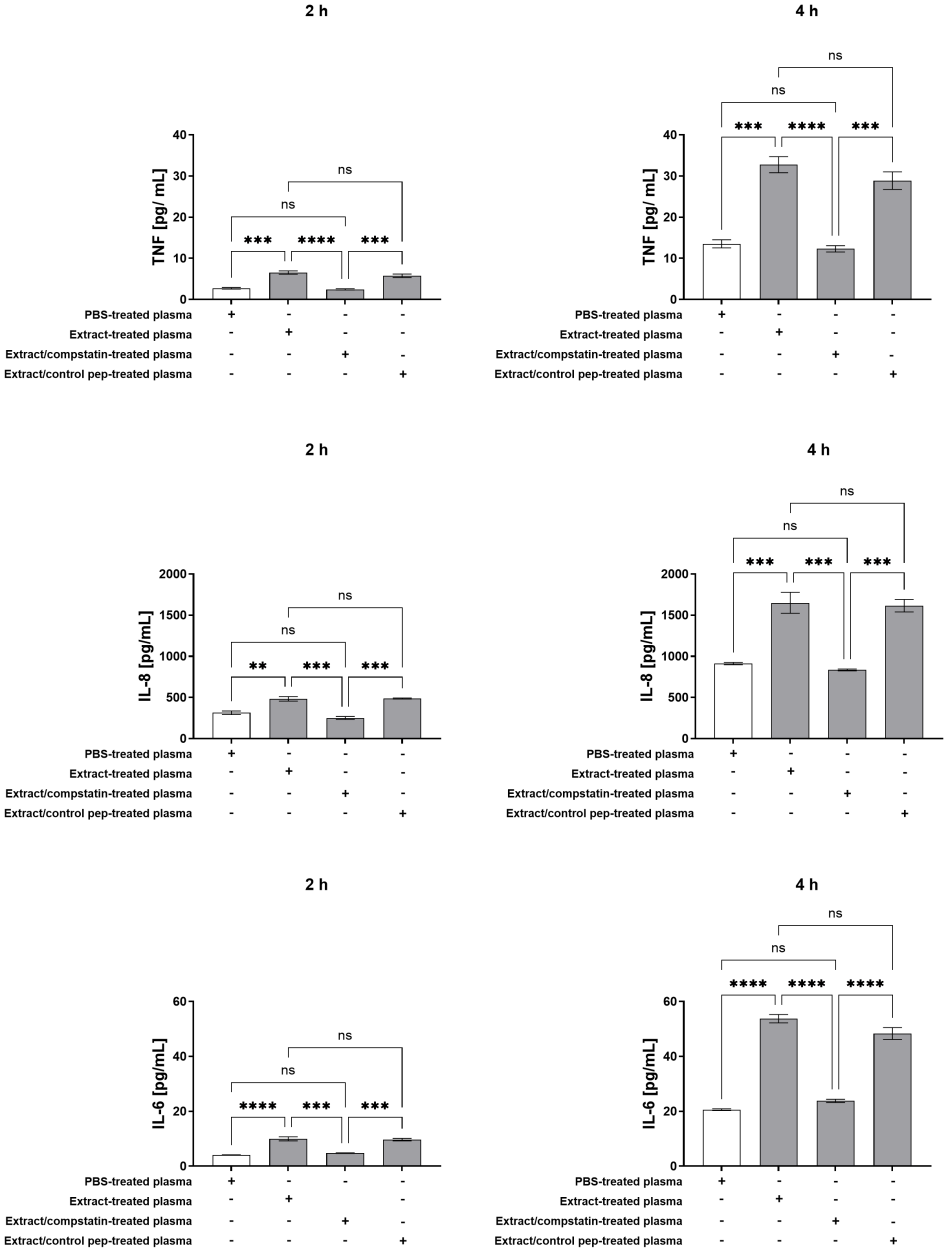
Production of TNF-α, IL-8, and IL-6 by Neutrophils Stimulated with Extract-Treated Plasma ± Compstatin. Neutrophils isolated from peripheral blood (1×10^5^ cells/well) were treated with plasma from whole blood treated with PBS, plasma from whole blood treated with extract, plasma from whole blood treated with extract in the presence of compstatin, and plasma from whole blood treated with extract in the presence of the control peptide for 2 and 4 hours at 37°C in a 5% CO_2_ incubator. After stimulation, TNF-α, IL-8 and IL-6 production was determined in the culture supernatants using a Cytometric Bead Array. Results are presented as mean ± SEM of triplicates from three independent experiments. **p < 0.01, ***p < 0.001, ****p < 0.0001.

**Figure 5:**
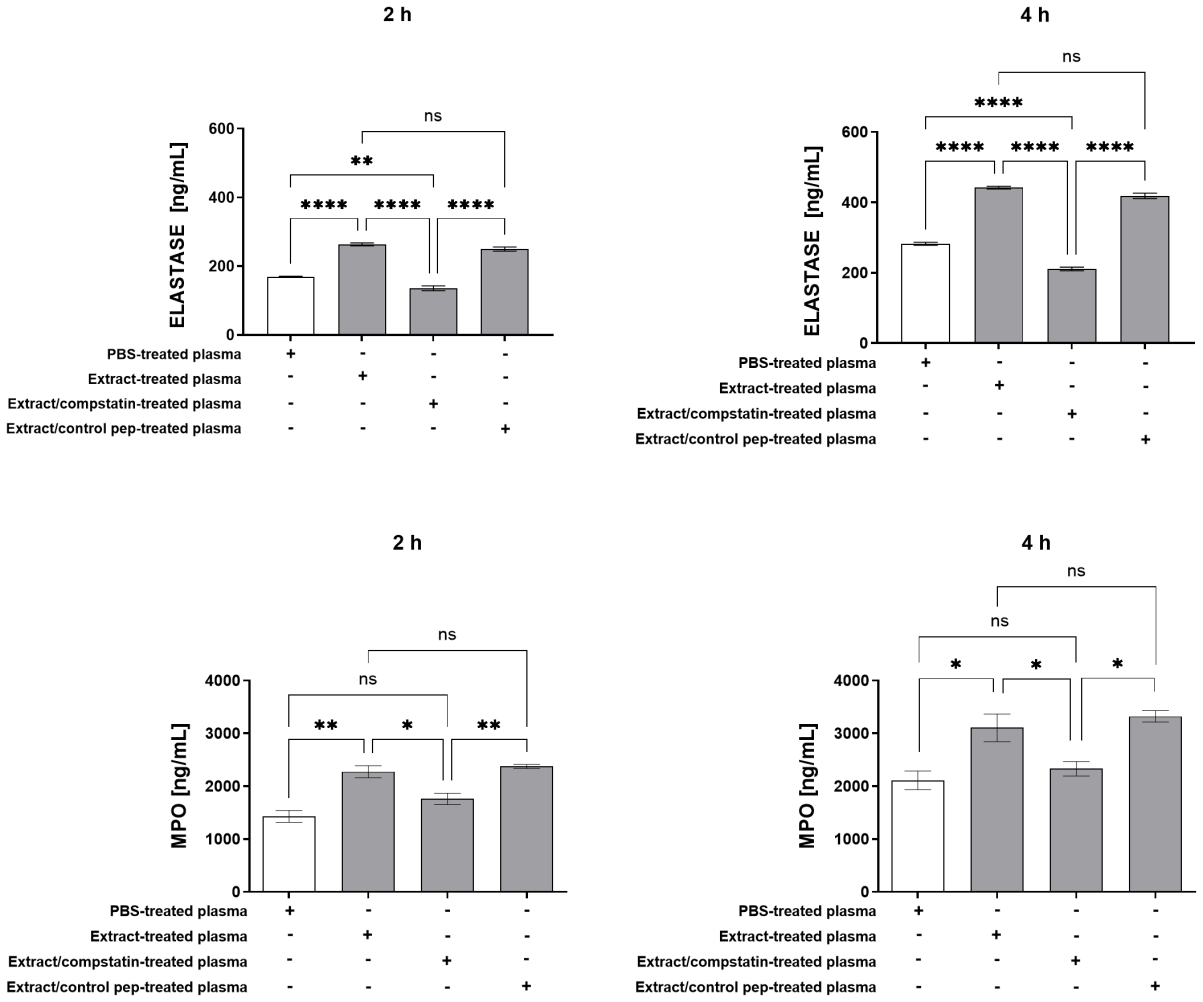
Elastase and MPO Production by Neutrophils Stimulated with Extract-Treated Plasma ± Compstatin. Neutrophils isolated from peripheral blood (1×10^5^ cells/well) were treated with plasma from whole blood treated with PBS, plasma from whole blood treated with extract, plasma from whole blood treated with extract in the presence of compstatin, and plasma from whole blood treated with extract in the presence of the control peptide for 2 and 4 hours at 37°C in a 5% CO_2_ incubator. After stimulation, elastase and myeloperoxidase production was determined in the culture supernatants by ELISA. Results are presented as mean ± SEM of triplicates from three independent experiments. *p < 0.05, **p < 0.01, ****p < 0.0001.

As shown in **Figure 4**, plasma from whole blood treated with pararama hair extract stimulated neutrophils to produce TNF-α, IL-8, and IL-6 after 2 and 4 hours. Inhibition of the complement pathway at C3 with compstatin effectively suppressed this inflammatory response, restoring mediator levels to baseline, similar to plasma from PBS-treated whole blood. Elastase was also detected in neutrophil culture supernatants after 2 and 4 hours of stimulation with plasma from extract-treated whole blood. Compstatin significantly inhibited elastase release at both time points, with levels after 4 hours even lower than those seen with plasma from PBS controls (**Figure 5**). MPO release was significantly increased under the same conditions, and compstatin fully restored baseline levels at both 2 and 4 hours (**Figure 5**). The control peptide did not induce any significant changes and showed values comparable to the extract-only condition, confirming that the effects of compstatin were specific to C3 inhibition.

Analysis of the presence of neutrophil extracellular traps (NETs), assessed by quantifying MPO-DNA complexes in culture supernatants, revealed no detectable levels following stimulation with plasma derived from either PBS or pararama hair extract-treated whole blood samples (data not shown).

### 3.3 Inhibition of the C5a-C5aR1 axis reduces inflammatory mediator production induced by pararama hair extract-treated plasma

Given that plasma from whole blood treated with pararama hair extract stimulated the production of multiple inflammatory mediators by neutrophils, and that this response was attenuated by complement system inhibition at the C3 level via compstatin, we further investigated the specific role of the C5a-C5aR1 axis in this inflammatory cascade. To this end, neutrophils were pretreated with PMX205, a selective C5aR1 antagonist, and subsequently stimulated with plasma derived from extract-treated whole blood. Cytokine and granular enzyme levels were then quantified in the culture supernatants.

**Figure 6:**
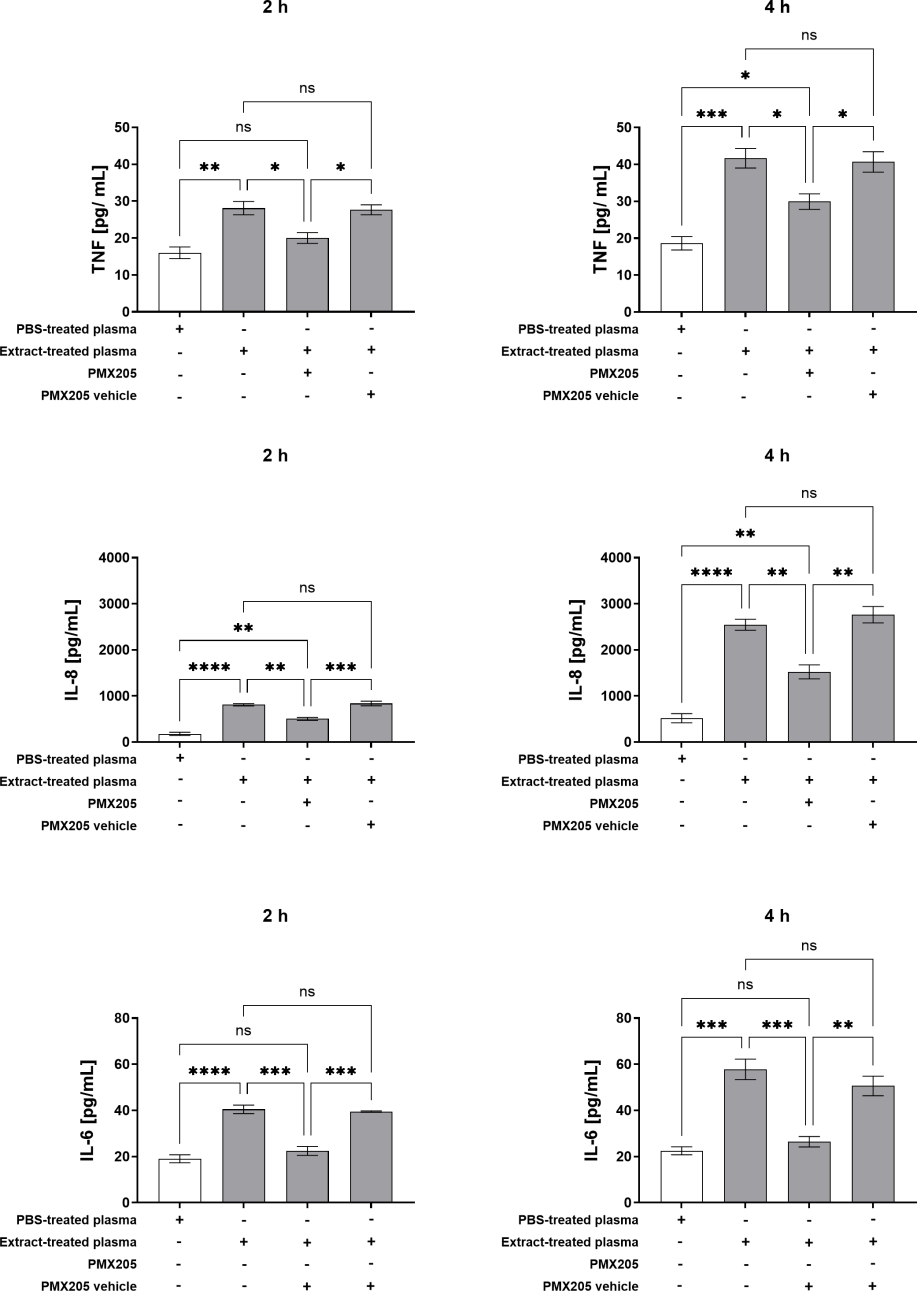
Production of TNF-α, IL-8, and IL-6 by Neutrophils Stimulated with Extract-Treated Plasma ± PMX205. Neutrophils isolated from peripheral blood (1×10^5^ cells/well) were pretreated with PMX205 or its vehicle (5% glucose) and stimulated with plasma from whole blood treated with PBS, or plasma from whole blood treated with extract, for 2 and 4 hours at 37°C in a 5% CO_2_ incubator. Following stimulation, TNF-α, IL-8 and IL-6 production was determined in the culture supernatants using a Cytometric Bead Array. Data are presented as mean ± SEM of triplicates from three independent experiments. *p < 0.05, **p < 0.01, ***p < 0.001 and ****p < 0.0001.

**Figure 7:**
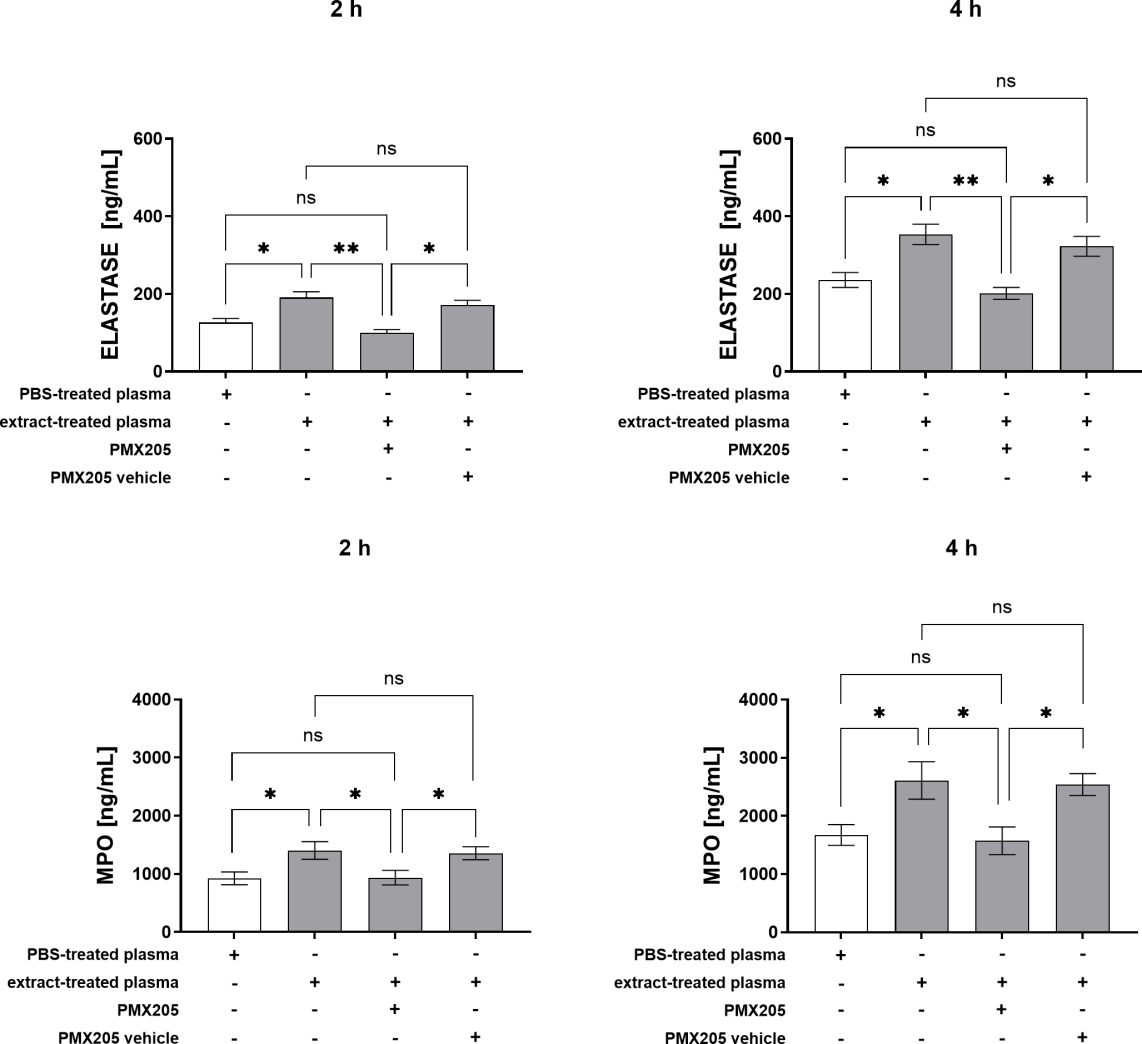
Elastase and MPO Production by Neutrophils Stimulated with Extract-Treated Plasma ± PMX205. Neutrophils isolated from peripheral blood (1×10^5^ cells/well) were pretreated with PMX205 or its vehicle (5% glucose) and stimulated with plasma from whole blood treated with PBS, or plasma from whole blood treated with extract, for 2 and 4 hours at 37°C in a 5% CO_2_ incubator. Following stimulation, elastase and myeloperoxidase production was measured in culture supernatants by ELISA. Data are presented as mean ± SEM of triplicates from three independent experiments. *p < 0.05 and **p < 0.01.

As shown in **Figure 6**, blockade of the C5a-C5aR1 axis with PMX205 markedly reduced TNF-α production induced by plasma from extract-treated whole blood at both 2 and 4 hours post-stimulation. A similar reduction in IL-8 levels was also observed at these time points. C5aR1 inhibition effectively normalized IL-6 levels to those observed in control conditions. Quantification of neutrophil granular contents revealed that stimulation with plasma from extract-treated whole blood significantly increased myeloperoxidase (MPO) and elastase release after 2 and 4 hours. However, pretreatment with PMX205 markedly reduced the release of both MPO and elastase, restoring their levels to baseline values comparable to those in control cultures. These results underscore the crucial role of complement activation, particularly via the C5a-C5aR1 pathway, in driving the neutrophilic inflammatory response triggered by plasma components resulting from pararama hair extract stimulation (**Figure 7**).

## 4 Discussion

This study provides novel insights into the mechanisms by which the extract from *Premolis semirufa* caterpillar hairs activates human neutrophils and amplifies inflammatory responses, contributing to the pathogenesis of pararamosis. Our findings demonstrate that the extract triggers a robust activation of neutrophils, both directly and via complement-dependent mechanisms, leading to the release of a broad spectrum of inflammatory mediators and effector molecules.

Direct exposure of isolated human neutrophils to the pararama hair extract resulted in a significant increase in the secretion of pro-inflammatory cytokines (including IL-6 and TNF-α) and chemokines (such as CXCL8/IL-8), as well as the granular enzymes myeloperoxidase (MPO) and elastase. These findings are especially significant given the role of neutrophils in inflammatory joint diseases such as rheumatoid arthritis and osteoarthritis. Neutrophil-derived enzymes, including elastase and myeloperoxidase, are elevated in the synovial fluid of affected joints, with MPO also detectable in plasma and urine, underscoring its potential as a biomarker for these conditions (55–64). Moreover, our results are consistent with previous observations in murine models, where repeated administration of the extract led to intense local inflammation, marked neutrophil infiltration, and elevated cytokine production (11, 12). The robust release of MPO and elastase further underscores the potential of neutrophil activation to contribute to tissue injury and the transition from acute inflammation to chronic joint damage, as described in clinical cases of pararamosis (7, 8).

The extract also induced the formation of neutrophil extracellular traps (NETs), as evidenced by increased levels of MPO-DNA complexes in the culture supernatant, although to a lesser extent than PMA, a classical NET-inducing agent (65–67). Interestingly, plasma derived from whole blood treated with pararama hair extract, which is rich in complement activation products, also induced neutrophil activation, demonstrating similar effects, including increased cytokine production and degranulation. The use of the central complement inhibitor, compstatin, to shut down complement activation, and the C5aR1 inhibitor PMX205, to block C5a interaction with its receptor, markedly suppressed these responses, underscoring the essential role of complement in this activation process. In contrast, plasma treated with the extract did not induce the formation of NETs. We hypothesize that this lack of NET formation in the plasma-treated condition may be due to the degradation of NETs by plasma-derived DNAses, preventing their detection. This hypothesis aligns with previous reports describing NET degradation by DNases in human fluids (65, 68–70).

While C3a and sTCC fragments can modulate neutrophil behavior, their effects are weaker compared to C5a (71). For example, C3a does not induce significant changes in neutrophil ROS generation or phagocytic activity, unlike C5a (72, 73). Notably, we observed that PMX205, unlike compstatin, did not fully restore TNF-α and IL-8 levels to baseline, suggesting that additional complement products may contribute to neutrophil activation.

The complex interplay between complement activation and neutrophil function is central to inflammatory regulation. Neutrophils respond robustly to the complement-derived anaphylatoxin C5a, and also directly promote complement activation. Halbgebauer et al. (2018) (74) demonstrated that neutrophil-derived NETs from trauma patients contain complement proteins (C1q, C3, properdin), which may serve as scaffolds for local complement activation. Neutrophil proteases like elastase modulate complement components and receptor signaling (75–77), while neutrophil fragmentation activates the alternative and lectin complement pathways, increasing plasma markers such as C4bc, C3bBbP, C5a, and sC5b-9 (78). Complement fragments, especially C5a and C5a des-Arg, rapidly induce phenotypic and functional changes in neutrophils, including membrane depolarization, intracellular pH shifts, glucose uptake, ROS production, and modulation of surface markers. This highlights the C5a–C5aR1 axis as a key mediator in diseases like sepsis and rheumatoid arthritis (21, 22, 71, 79, 80). Complement-neutrophil interactions also contribute to vascular inflammation by promoting calcium influx, cell adhesion, and leukocyte migration through regulation of adhesion molecules and enhancing neutrophil-endothelial interactions (81). Microfluidic studies confirm C5a as a potent neutrophil chemoattractant, whereas C3a has minimal effect (82). Selective C5a–C5aR1 blockade (e.g., avacopan, RA101295) reduces chemotaxis while preserving phagocytosis, underscoring its therapeutic potential (82–85). Additionally, sublytic concentrations of the terminal complement complex (sTCC) modulate immune cell function and inflammatory signaling, adding further complexity to complement–neutrophil crosstalk (86–89).

Beyond neutrophil activation, the complement system plays a crucial role in osteoarthritis (OA) pathogenesis. Wang et al. (2011) (90) demonstrated upregulation of complement components (C3, C5, C7, C9) and downregulation of regulators like factor H and clusterin in OA synovial tissues, fostering complement activation. Sublytic membrane attack complex (MAC) deposition on chondrocytes activates ERK1/2 and increases expression of MMP-13, CCL2, CCL5, COX-2, and prostaglandin E2, promoting cartilage degradation and chronic inflammation. Earlier studies by Cooke et al. (1980) (91) and Corvetta et al. (1992) (92) confirmed MAC deposition in OA joint tissues. Murine models deficient in C5 or C6 were protected from joint damage and synovitis, while CD59a-deficient mice showed worsened OA, highlighting MAC’s pathogenic role (90). Therapeutic use of CR2-fH fusion proteins mimicked complement deficiency protection, reinforcing MAC’s involvement in inflammatory joint disease (93). These findings tie into neutrophil participation in inflammatory joint diseases, where neutrophil enzymes elastase and MPO are elevated in synovial fluid, and MPO is also detectable in plasma and urine, serving as a biomarker (55–58, 60–64).

In conclusion, our findings show that pararama hair extract activates neutrophils both directly and via complement-dependent mechanisms, as demonstrated by the effects of complement inhibitors. This activation leads to the release of inflammatory mediators and NETs, contributing to the persistent inflammation seen in pararamosis. These results underscore the importance of neutrophils in the disease process and suggest that complement-targeted therapies could be valuable for controlling inflammation and tissue damage in affected individuals.

## Data Availability

The original contributions presented in the study are included in the article/**Supplementary Material**. Further inquiries can be directed to the corresponding author.
