# Supplementary material for: Complement-mediated and direct activation of human neutrophils induced by Premolis semirufa caterpillar toxins
Source: Front Immunol. 2026 Jan 2;16:1706235. doi: 10.3389/fimmu.2025.1706235 (PMC12807961; doi:10.3389/fimmu.2025.1706235)
Supplement: Supplementary file 1 [file DataSheet1.docx]

**
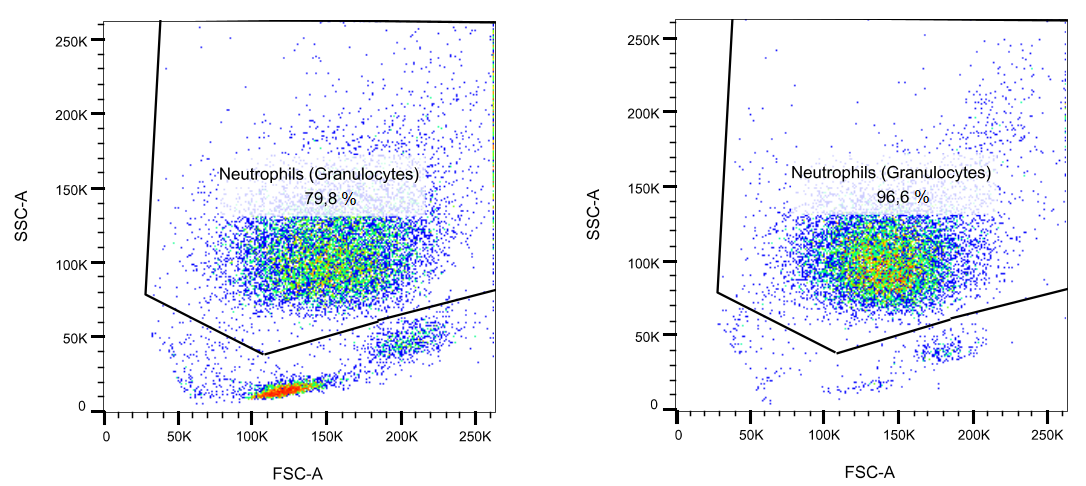
**

**Supplementary Figure 1. Flow cytometry analysis of neutrophil (granulocyte) populations before and after Ficoll-Paque PLUS separation. Neutrophils were isolated from peripheral blood by dextran sedimentation of erythrocytes, followed by hypotonic lysis of residual red cells. The resulting cell suspension was subjected to Ficoll-Paque PLUS density gradient separation. Representative dot plots show neutrophil (granulocyte) gating based on forward scatter (FSC) and side scatter (SSC). Left: sample before Ficoll-Paque PLUS separation. Right: sample after Ficoll-Paque PLUS separation, showing enrichment of neutrophils (granulocytes). Percentages indicate the proportion of neutrophils (granulocytes) within the analyzed cell population after debris exclusion.**
